# Towards Increasing the Clinical Relevance of In Silico Methods to Predict Pathogenic Missense Variants

**DOI:** 10.1371/journal.pcbi.1004725

**Published:** 2016-05-12

**Authors:** David L. Masica, Rachel Karchin

**Affiliations:** 1 Department of Biomedical Engineering and The Institute for Computational Medicine, The Johns Hopkins University, Baltimore, Maryland, United States of America; 2 Department of Oncology, The Johns Hopkins University School of Medicine, Baltimore, Maryland, United States of America; National Cancer Institute, UNITED STATES

## Overview

As genetic sequencing throughput continues to accelerate, so does the accumulation of variants of unknown clinical significance. The great majority of these variants cause amino acid substitutions (cSNVs) in protein sequence. The need to interpret these variants continues to motivate development of better in silico bioinformatic methods. Despite the development of dozens of such methods over the past 15 years, clinically relevant prediction accuracy remains elusive. Here, we present some recent progress and shortcomings in the development of bioinformatics missense variant classifiers, and we argue for the increased use of endophenotypes. Endophenotypes are quantitative measurements that are correlated with phenotypes via shared genetic causes (e.g., enzyme catalytic activity, serum cholesterol or glucose level, volumetric lung capacity). In many cases, endophenotypes are more directly influenced by genetic variation, increasing their power to detect genotype-endophenotype associations relative to genotype-phenotype associations. The data required to train and benchmark bioinformatic methods to predict endophenotype from cSNVs and other variant types is increasingly available and could be made widely available by concerted community effort to enhance locus-specific and disease variant databases. We highlight some currently available data and present results from published bioinformatics studies that use endophenotypes.

## Introduction

The 21st century has thus far been marked by a heroic effort in genomic science and technology. If not yet upon us, the age of personalized genomic medicine appears imminent. Deriving medically relevant insight from these advances requires the ability to interpret the genetic variation and similarity observed in the population. One approach for interpreting genetic variation is the use of bioinformatics methods; simply put, these approaches are unified by a reliance on molecular/biological information (DNA, RNA, and protein sequence and annotation, protein structure, etc.) [1–4]. Many bioinformatics classifiers, primarily focused on amino acid substitution variants (cSNVs), have been and continue to be developed, typically achieving classification accuracies much better than random and thus supporting the use of molecular information [5–9]. These methods appear, however, to have reached a performance bottleneck; currently realized limits in performance all but forbid the consultation of bioinformatics cSNV classifiers in clinical settings. This bottleneck might be, in part, the result of simplifying assumptions inherent to the prediction of qualitative, dichotomous, or categorical phenotypes from individual missense variants.

A complementary and beneficial strategy could be greater use of endophenotypes, quantitative measurements that are related to phenotypes via shared underlying genetics [10,11]. Endophenotypes can include phenomena at diverse biological scales; some examples include protein catalytic rate or melting temperature (stability), cell growth rate, and blood pressure. In this perspective we make a case for the increased use of endophenotypes, beginning with a brief overview of in silico bioinformatics methods for assessing phenotypic impact of cSNVs and the performance reported in recently published comparative studies. We compare the utility of endophenotypes and phenotypes in the context of *CFTR* cSNVs in cystic fibrosis, and of *LDLR* cSNV impact on cardiovascular-related diseases such as familial hypercholesterolemia. We also provide examples of bioinformatics methods that have been used to predict endophenotypes from cSNVs.

## Bioinformatics Classifiers of Phenotype: Background and Performance

Bioinformatics methods to predict the pathogenicity of cSNVs typically utilize gene/protein sequence, protein structure, annotation, or some combination of the three [1–4]. Table 1 shows 13 methods tested in up to five independent large-scale studies, one from each of the past five years [5–9]. Each of the five independent studies used large sets of putatively neutral and pathogenic variants, and three of these studies considered two such datasets; Olatubosun et al. used the Protein Mutant Database (PMD) twice, the second time using only a subset of variants defined as being reliably predicted by their own PON-P method. Criteria for inclusion in this table was that an independent group directly compared the method with other methods; for the fairest comparison, if an author included an assessment of their own method, that assessment is not shown in Table 1. Each of the five studies included the statistical sensitivity, specificity, and accuracy, or included the relevant contingency table; therefore, we present these three performance metrics for each of the tested method-dataset combinations (Table 1). While these performance metrics alone cannot provide a truly comprehensive estimate of classifier performance, they do facilitate a reasonable comparison of methods and a general assessment of method utility.

**Table 1:** Five years of independent testing of cSNV variant classifiers.

	Thusberg et al. 2011	Olatubosun et al. 2012	Shihab et al. 2013	Rapakoulia et al. 2014	Dong et al. 2015
	**+** 19,335 SwissProt & LSDBs - 21,170 dbSNP	+ 902 PMD - 858 PMD	+ ~23,000 SwissVar - ~34,000 SwissVar	+ 8,871 SwissProt - 24,342 dbSNP	+ 120 Curated by author - 124 CHARGE seq. proj.
	+ 3,594 LSDBs - 3,594 dbSNP	Subset reliable at 0.95		+ HumVar 22,196 - HumVar 21,119	+ 6,279 VariBench II - 13,240 VariBench II
**SIFT**	68%, 62% (65%) 65%, 62% (64%)	83%, 41% (62%) 83%, 48% (66%)	67%, 82% (74%)	38%, 81% (69%) 37%, 90% (63%)	68%, 75% (71%) 75%, 69% (71%)
**PolyPhen-2**	73%, 70% (71%) 62%, 69% (66%)	84%, 40% (62%) 81%, 50% (66%)	86%, 61% (73%)	85%, 69% (73%) 87%, 72% (80%)	92%, 59% (78%) 88%, 49% (63%)
**Panther**	77%, 76% (76%) 52%, 75% (64%)	NA NA	62%, 75% (68%)	31%, 90% (74%) 31%, 89% (59%)	38%, 94% (66%) NA
**PhD-SNP**	63%, 79% (71%) 40%, 79% (60%)	67%, 61% (64%) 69%, 74% (72%)	66%, 74% (70%) 77%, 79% (78%)	NA NA	70%, 83%, (77%) NA
**SNAP**	88%, 56% (72%) 74%, 56% (65%)	73%, 54% (63%) 72%, 65% (68%)	NA	NA NA	53%, 70% (61%) NA
**SNPs&GO**	71%, 92% (82%) 73%, 92% (82%)	NA NA	76%, 89% (82%)	NA NA	55%, 94% (75%) NA
**MutPred**	85%, 78% (81%) 71%, 77% (74%)	NA NA	90%, 90% (90%)	NA NA	74%, 81% (77%) NA
**Mutation Assessor**	NA NA	NA NA	NA	73%, 88% (84%) 77%, 84% (80%)	70%, 80% (74%) 73%, 74% (74%)
**nsSNAPAnalyzer**	61%, 58% (60%) 58%, 56% (57%)	NA NA	NA	NA NA	NA NA
**MutationTaster**	NA NA	NA NA	NA	NA NA	94%, 74% (86%) 91%, 49% (64%)
**FATHMM**	NA NA	NA NA	NA	NA NA	55%, 91% (75%) 86%, 82% (83%)
**CONDEL**	NA NA	NA NA	NA	NA NA	71%, 73% (72%) 71%, 71% (71%)
**CADD**	NA NA	NA NA	NA	NA NA	79%, 74% (77%) 75%, 65% (69%)

Selected studies, one from each of the past five years, comparing multiple bioinformatic variant classifiers on large datasets of putatively pathogenic and benign variants. To avoid any potential bias, tests of an author’s own method, if present, were excluded. For each entry, results are given as *sensitivity*, *specificity* (*accuracy*). Variants datasets used as the pathogenic or disease-causing class are indicated by a plus sign (+) and the neutral or benign class with a negative sign (-). Entries with multiple lines indicate that two unique datasets were used for benchmarking. Both datasets used in Olatubosun et al. 2012 were the same, except that variants in the second set were filtered to meet a 95% prediction confidence using their own PON-P method. PMD is the Protein Mutant Database; NA indicates the method was not tested in the corresponding benchmark.

SIFT [12] and PolyPhen-2 [13] are two of the most commonly cited and used methods to predict disease phenotype from cSNVs. Across the five independent tests in Table 1, accuracies achieved by both methods typically ranged from the mid-60s to mid-70s. Another important result for these two methods is that the trade off between sensitivity and specificity varies significantly among benchmarks. For instance, SIFT and PolyPhen-2 both had high sensitivities in the Olatubosun et al. benchmark, and SIFT had disproportionately high specificity in both of the Rapakoulia et al. benchmarks. A similar variability in sensitivity and specificity across benchmarks, as well as accuracy range, was reported for SNAP [14]. PhD-SNP [15] was tested in four of the five studies, achieving accuracies comparable to most methods presented in Table 1, with a reasonable balance between sensitivity and specificity in all tests. Panther [16] was also tested in four of the studies, achieving accuracies similar to SIFT, PolyPhen-2, PhD-SNP, and SNAP, but typically realizing better gains in specificity than sensitivity. SNPs&GO [17] was also skewed toward specificity, realizing a slightly higher accuracy than SIFT, PolyPhen-2, Panther, and SNAP. MutPred [18] and Mutation Assessor [19] were tested in three and two of the studies, respectively; these methods achieved relatively high accuracies and balanced sensitivities and specificities (Table 1).

## Origins and Implications of Variant Classifier Performance

Results for some of the method-benchmark combinations presented in Table 1 are promising. However, for most methods—in particular, those subject to the greatest scrutiny—reported performance is inconsistent across benchmarks, including highly unbalanced sensitivity and specificity. Bioinformatics methods are not currently recommended for medical decisions that require variant interpretation [20]. We believe that these perceived methodological shortcomings might, in part, arise from assumptions inherent in the prediction of qualitative biological phenomena from individual genetic variants. Genome-wide association studies (GWAS) have thus far indicated that the vast majority of genetic variation in complex diseases likely impact gene regulation and have low effect size [21,22]. Similarly, pedigree studies have recovered relatively few high-penetrance genes/variants [23,24]. And recent whole-genome and targeted sequencing efforts are revealing that healthy individuals, on average, harbor tens of so-called “disease alleles,” including many in the homozygous state [24,25]. Thus, bioinformatics predictors are challenged with predicting disease from individual variants, yet individual variants appear to rarely carry significant disease liability. This is particularly true of cSNVs, which on average have a more subtle effect on disease than truncating variants [26]. And because “disease alleles” used for classifier training and benchmarking may be relatively common among healthy individuals, expectations about the relevance of subsequent predictions should be tempered.

**Fig 1:**
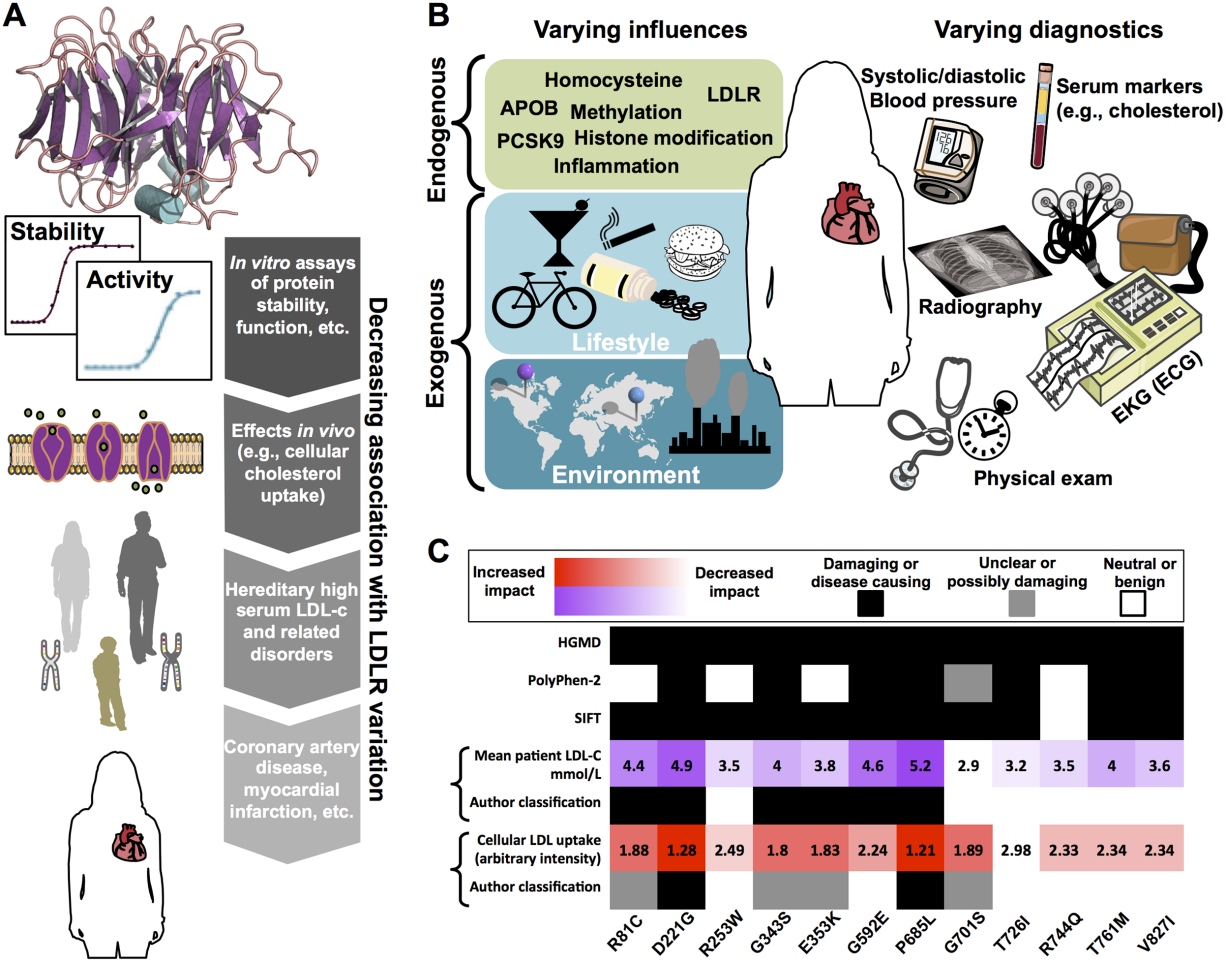
How context can influence the impact and inferred impact of LDLR variation on different experimental and clinical parameters. LDLR variation is likely to have the largest observable and reproducible impact on parameters most directly influenced by the protein (*A*). For instance, if a variant produces effects downstream from the protein, then protein structure and function are likely perturbed. Further downstream, cellular LDL uptake could be modulated, which can increase risk for familial hypercholesterolemia, which might appreciably increase risk for heart disease or heart attack. Liability for complex cardiovascular diseases is influenced by many endogenous and exogenous factors other than LDLR mutation (*B*). Furthermore, diagnoses can result from a varied combination of clinical and laboratory diagnostics, which can result in differential or conflicting diagnoses (*B*). In *C*, cellular studies, pedigree studies, a disease mutation database, and popular bioinformatics methods are used to classify LDLR variants as disease causing or benign. On the heatmap, black and white indicate a classification of disease causing and benign, respectively, for different classification methods (gray indicates an intermediate or unclear classification). Mean patient LDL-c concentration from pedigree studies (purple) and cellular LDL uptake (red) shown with darker colors indicating more severe impact (numbers indicate published values).

Fig 1 illustrates some factors that may confound bioinformatic cSNV disease phenotype prediction, using as an example the impact of *LDLR* (low-density lipoprotein receptor) cSNVs on cardiovascular-related diseases. LDLR is a protein localized to the plasma cell membrane that enables endocytosis of low-density lipids into cells and controls plasma concentration of low-density lipid cholesterol (LDL-c). Changes in LDLR protein stability and activity, which are most closely related to cSNVs in LDLR, are likely to be easiest to predict (Fig 1A). The further downstream an effect is from LDLR function (a diagnosis of coronary artery disease, for instance), the more likely additional factors are to influence the effect. These influences can include other endogenous inputs (e.g., genetic and epigenetic factors) as well as exogenous factors such as lifestyle and environment (Fig 1B). Thus, a cSNV that perturbs LDLR stability and/or activity will not necessarily cause disease. This is of practical importance because bioinformatics predictors rely heavily on features of protein sequence, structure, and function, but are typically tasked with predicting disease. Further complicating is the fact that diagnosis of a particular disease phenotype can result from different diagnostic tests, combination of tests, and varied interpretation of test results (Fig 1B). In contrast, measured effects in cellular assays and diagnostics such as high serum LDL cholesterol are closer to the LDLR protein stability and activity required for normal function, and they are not in theory confounded by subjective factors implicit to associating cSNVs with disease diagnosis.

Fig 1C shows 12 LDLR cSNVs that were common to multiple studies with published variant-specific mean patient LDL-c level, cellular assays of LDL uptake, bioinformatics prediction with SIFT and Polyphen-2, and curation in the Human Gene Mutation Database (HGMD) [27–29]. Supporting the idea that endophenotypic measurements may be more consistent than disease phenotype categories, the clinical LDL-c concentration and experimental LDL-uptake assays from two different laboratories show the expected negative correlation (Pearson correlation = −0.69; *p*-value = 0.013). (Normal LDLR uptake of LDL lowers plasma LDL-c levels.) However, the assignment of each cSNV to a disease phenotypic category differs depending on the selected endophenotype studied and is often in disagreement with HGMD. As in the independent benchmarking studies (Table 1), the accuracy and specificity/sensitivity trade-off of the bioinformatics classifiers depends on which categorization is considered to be the gold standard.

Using clinical diagnostics, experimental assays, and biomedical literature to derive gold-standard mutation databases is reasonable and commonplace. But, given that most variants, and cSNVs in particular, are low effect and incompletely penetrant, the disagreement among these potential gold standards should be unsurprising. Therefore, it becomes unclear how and to what extent the disappointing performance of bioinformatics methods should be interpreted, given that many cSNVs could reasonably be placed in multiple phenotypic categories. In contrast, the continuous-valued cellular LDL-uptake and serum LDL-c measurements rely only on accurate determination, rather than varied and arbitrary thresholds for classification. These types of endophenotypic measurements represent a practical and useful target for prediction and help circumvent some potentially unreliable presuppositions currently associated with bioinformatics cSNV prediction.

## Endophenotypes: An Alternative and Complementary Framework

The term “endophenotype” was coined in 1966 to distinguish the observable, external phenotype (exophenotype) from internal or microscopic traits [30]. In 1972, Gottesman and Shields reintroduced the term in the context of schizophrenia to describe internal phenotypes discoverable by biochemical assays or microscopic examination [31]. Used infrequently over the next several decades, the word “endophenotype” experienced quite a renaissance after the publication of a 2003 review article by Gottesman and Gould [11]. Endophenotypes are most often explicitly used in the context of psychiatric disorders such as schizophrenia or bipolar disorder, but the endophenotype concept has been applied in the context of many diseases, including obesity [32], diabetes [33], osteoporosis [34], heart disease [27–29,35], hypertension [36], phenylketonuria [37], and cystic fibrosis [38–40]. Requirements of heritability and co-segregation have been suggested in order for a quantitative trait to be considered a true endophenotype [10,11,41]. For this perspective, we use a broad definition of endophenotype; in short: endophenotypes are quantitative traits that are associated with qualitative traits (phenotypes) via shared genetic influences. Importantly, endophenotypes include the quantitative risk factors that are often used to diagnose and define disease (e.g., serum metabolite concentrations, blood pressure, sweat chloride), as well as molecular-scale phenomena such protein stability or catalytic rates.

**Fig 2:**
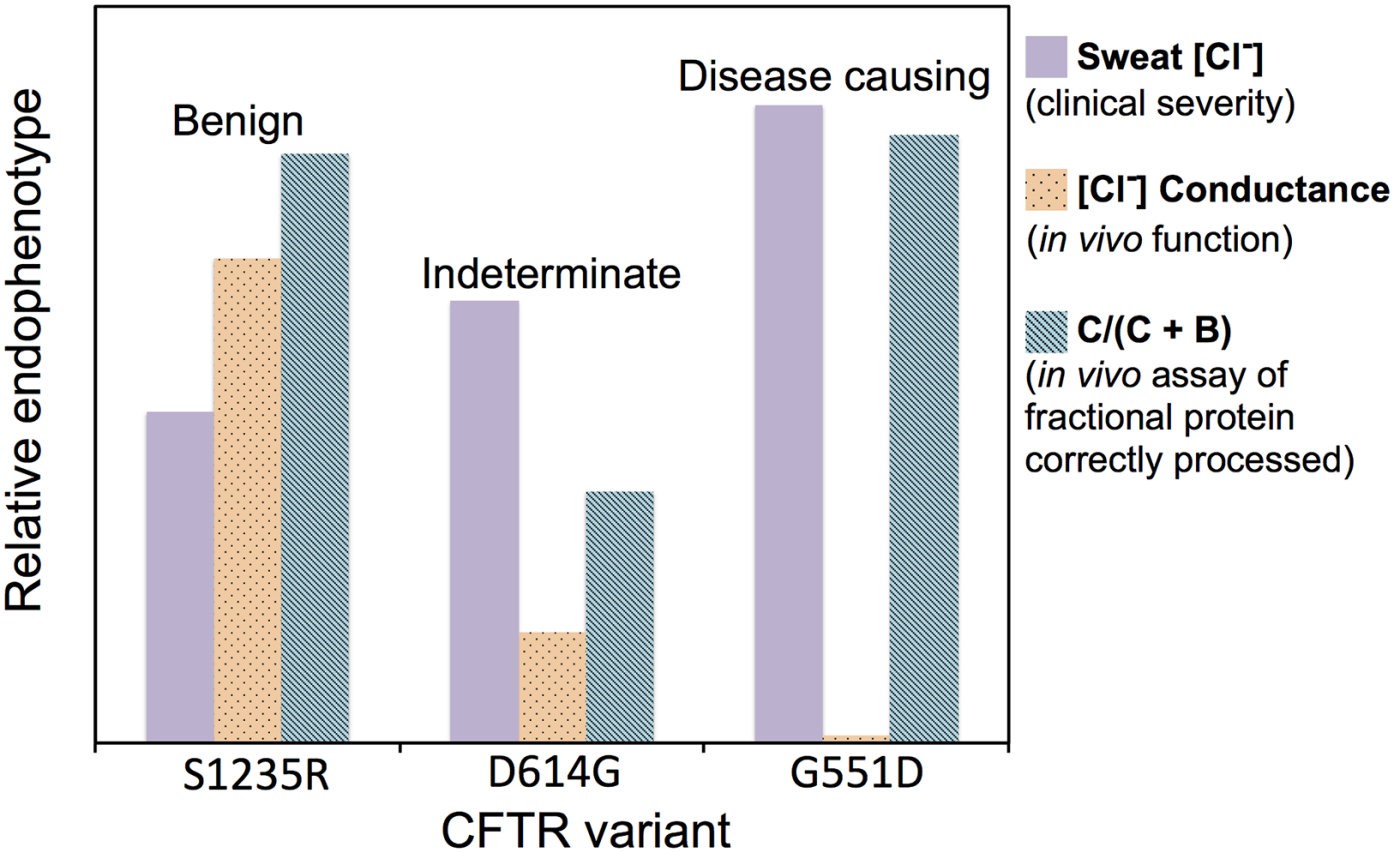
Some advantages of considering endophenotypes, relative to phenotypes, illustrated using three *CFTR* variants. Mean sweat chloride from individuals harboring the three variants (S1235R, D614G, and G551D), and results from two distinct in vivo experiments performed in cells expressing the variants. Increasing sweat chloride is associated with increasing disease severity, whereas in the two in vivo assays decreasing values correspond to decreasing protein function or abundance. Endophenotypes were scaled for purposes of presenting on a single chart, such that three sweat chlorides could be compared with one another, the three chloride conductance measurements could be compared with one another, etc.

By this definition, we believe that there are considerable benefits to bioinformatic approaches for predicting the genotype-endophenotype relationship, relative to that of the genotype-phenotype relationship: (1) Endophenotypes are closer to the level of gene action and protein function than the associated phenotypes, increasing the effect size and power to detect variant-endophenotype associations relative to that of the variant-phenotype associations. An example of this benefit is depicted in Fig 1A, where a cSNV in the LDLR protein is expected to have a more measureable effect on cellular LDL uptake than it would on the dichotomous prediction of having a heart attack or not. (2) By virtue of being qualitative, phenotypes rely on subjective and often arbitrary definitions. Although quantifying phenotypic descriptions is an active area of informatics research [42], the exact defining characteristics of a phenotype can change over time and be subject to disagreement among experts. Conversely, by virtue of being quantitative, endophenotypes should, in principle, rely only on accurate measurements. (3) Endophenotypes facilitate the ranking of biological states (e.g., disease severity) within the otherwise arbitrarily defined phenotypic categories. (4) The reliance on objective measurements rather than subjective definitions, along with the disposing of arbitrary thresholds for partitioning phenotypic categories, reduces data contamination and in turn benefits algorithmic training and benchmarking. (5) Endophenotypes can describe both severity and molecular mechanism with higher resolution than can phenotypes (Figs 1C and 2).

## The Benefit of Using Endophenotypes: An Illustrative Example

Endophenotypes can provide information that supplements phenotypic categories and increases their clinical utility, by pointing to specific disease severity and mechanism associated with a variant. This utility is illustrated by three cystic fibrosis transmembrane conductance regulator (*CFTR*) cSNVs shown in Fig 2, each having a distinct, clinically defined impact on cystic fibrosis disease liability (benign, indeterminate, and disease causing) [40].

The first endophenotype shown in Fig 2 is the continuous-valued clinical diagnostic of patient sweat chloride, which increases across the three phenotypic categories. The "sweat test" is considered the gold standard for diagnosing cystic fibrosis. Healthy individuals have sweat chloride concentrations of less than 30 to 40 mmol/L and a test reporting 60 mmol/L or greater most often results in cystic fibrosis diagnosis. As expected, the mean sweat chloride of patients harboring the benign cSNV S1235R is lower than that of patients harboring the indeterminate cSNV D614G, and is highest in patients with the disease-causing mutation G551D. The second endophenotype measures chloride conductance by in vivo cellular assays; transport of chloride ions through the plasma membrane of epithelial cells is a major function of CFTR. Defects in chloride conductance result in the mucus build-up associated with cystic fibrosis. Again as expected, chloride conductance negatively correlates with increasing disease severity. It is highest in patients with the benign cSNV, substantially lower in those with the indeterminate cSNV, and undetectable in those with disease-causing G551D. Lastly, there is a different and important trend for the third endophenotype, in vivo measurements of CFTR C-band B-band ratio or *C/(C + B)*, which measures the fraction of CFTR protein that is fully processed (glycosylated) and trafficked to the cell surface. Correct processing and trafficking is necessary but not sufficient for normal CFTR function. As expected, CFTR is correctly processed and trafficked for the benign cSNV, and for the indeterminate cSNV the fraction of correctly processed protein decreases. But surprisingly, for the disease-causing G551D mutation, the fraction of correctly processed protein is approximately equal to that found with the benign variant; it is this differential impact of G551D—benign with respect to post-translational processing and damaging with respect to proper chloride transport function—that facilitates the efficacy of the landmark, G551D-specific, cystic fibrosis drug Ivacaftor [43]. Importantly, G551D-mutant CFTR protein is processed and trafficked to the epithelial cell surface, but once there it exhibits decreased chloride conductance. Ivacaftor potentiates cells harboring the CFTR G551D mutation, restoring chloride conductance. Following clinical trials, Ivacaftor was approved in 2014 to treat patients harboring several other CFTR mutations characterized by high C-band B-band ratio and low chloride conductance [40,44].

Previously, it has been proposed that multiple phenotypic categories, spanning the range from the most benign to most pathogenic variants, might alleviate problems with potential subjectivity and over-simplification of disease/benign or disease/indeterminate/benign classifications [45]. Indeed, the American College of Medical Genetics (ACMG) has recently published guidelines that include a five-category standard for clinical variant interpretation in genes that cause Mendelian disorders [20]. The guidelines emphasize the limited clinical utility of the current generation of in silico bioinformatic prediction methods, in particular citing low specificity.

Bioinformatics methods designed to predict endophenotypes might be able to achieve greater accuracy and reliability than those designed to predict phenotypic categories. The assessment of such methods is not confounded by subjective choices about the number of phenotypic categories or the assignment of a variant to the correct category. In silico interpretation of a variant in terms of one or more endophenotypes may capture clinically important differences between variants that are placed in the same phenotypic category. For example, both the CFTR G551D mutation described above and CFTR N1303K are pathogenic according to ACMG standards, but the two mutations have different endophenotypic patterns. Unlike G551D, the N1303K mutation has both low C-band B-band ratio and low chloride conductance [40], indicating that the mechanism of CFTR dysfunction is different in N1303K. These differences are relevant to clinical decision-making, since Ivacaftor is indicated for G551D, while the newer drug Lumacaftor may be effective for mutations that impact post-translational processing of CFTR [44].

**Fig 3:**
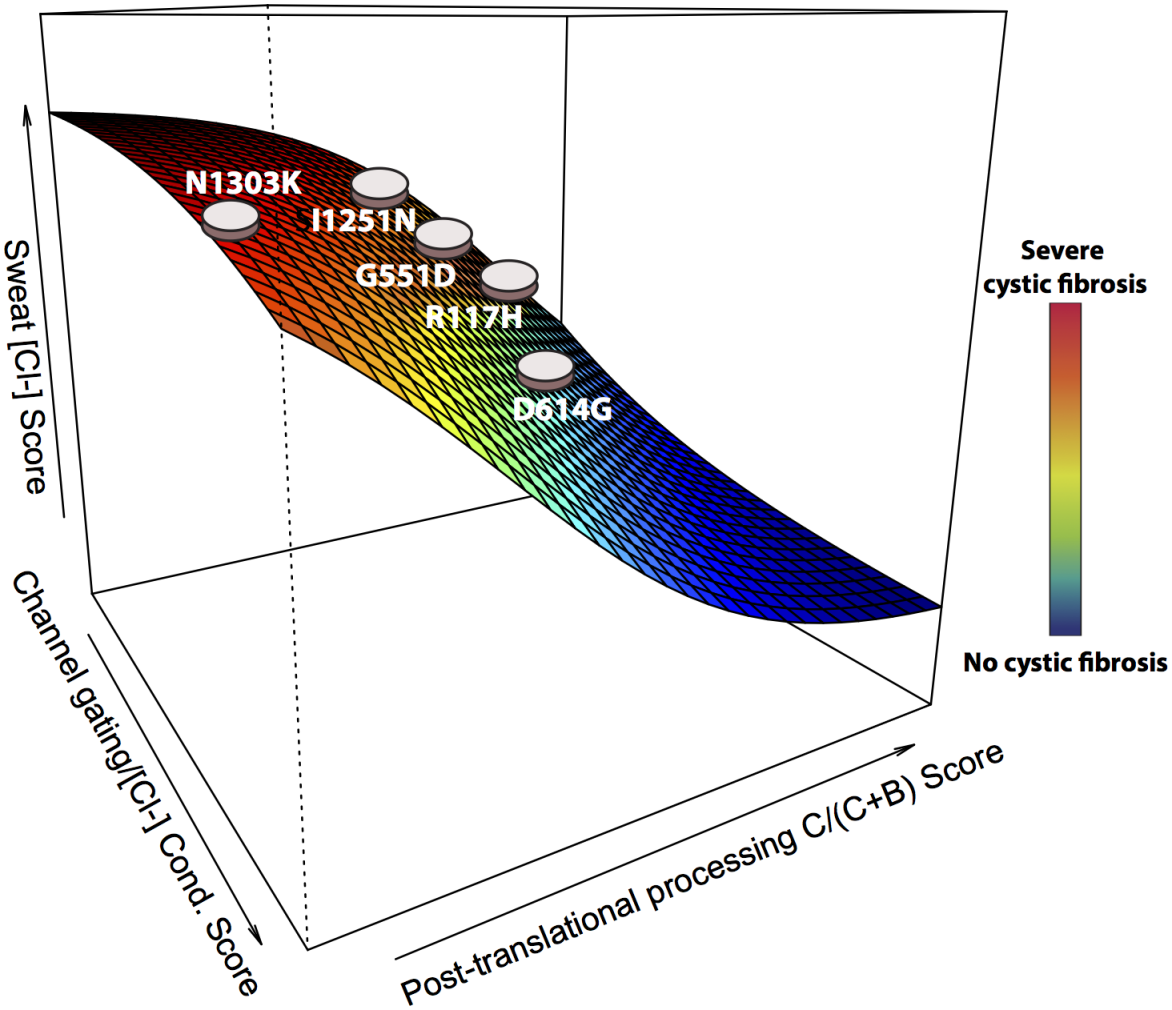
Hypothetical visualization of a multidimensional endophenotypic landscape for cystic fibrosis. Each cSNV can be represented as a point in a three-dimensional space of three endophenotypic scores relevant to cystic fibrosis disease: post-translational processing (glycosylation) and trafficking of the CFTR protein to the epithelial cell plasma membrane, an in vivo cellular assay of chloride conductance that measures channel gating, and chloride concentration in a diagnostic sweat test. Each point on the landscape can be interpreted with respect to disease severity, shown in the color bar to the right of the landscape.

The ability to visualize variants in a multidimensional landscape of several endophenotypes could be valuable for clinicians. Fig 3 shows a hypothetical landscape of cystic fibrosis severity along three orthogonal coordinates: post-translational process of CFTR, in vivo chloride conductance, and sweat chloride levels. Each cSNV can be represented as a point in the coordinate system, enabling clinical assessment of the relationship between the cSNV, disease severity, and multiple measures of disease mechanism.

## Bioinformatics Prediction of Endophenotypes

The output of most bioinformatic cSNV classifiers is a raw, continuous-valued score, which is transformed into the assignment of each cSNV to one of two or more categories. For instance, SIFT returns the probability that a protein will tolerate a cSNV, while PolyPhen-2 returns the probability that a cSNV is protein damaging. Although these methods were not developed to predict endophenotypes, their continuous-valued outputs could be used as informative endophenotypic correlates. This insight was utilized by Wettstein et al. to predict phenylalanine hydroxylase (PAH) activity and three phenylketonuria (PKU)-related endophenotypes as a function of PAH cSNVs [37]. In that study, the authors scored up to 834 PAH cSNVs using the SIFT, Polyphen-2, FoldX [46], and SNPs3D [47] packages. In the case of PAH activity, the authors found statistically significant correlation between FoldX score and PAH enzymatic activity, as well as for SNPs3D and PAH activity; neither SIFT or PolyPhen-2 scores were correlated with PAH activity. Similarly, the authors compared scores from the four methods with three PKU-related disorders (PKU, mild PKU, and mild hyperphenylalaninemia), and found significant association between continuous-valued FoldX, SNPs3D, and PolyPhen-2 scores and the three disease subtypes; SIFT scores and disease subtype were not significantly associated. This work shows that existing cSNV classifiers can be repurposed for predicting endophenotypic severity, as well as recovering categorical phenotype without necessarily requiring the use of arbitrary thresholds for partitioning scores.

The above-cited work of Wennstein et al. demonstrates the potential to repurpose existing cSNV classifiers; however, these existing methods are limited because they are agnostic to the endophenotype being predicted. This limitation is important, because different variants in the same gene can affect disease via distinct mechanisms (Fig 2), or be causal of different diseases entirely (e.g., *NF1* mutation can drive cancer or cause neurofibromatosis). We hypothesize that detecting the subtle biological underpinnings that converge to influence a particular mutation-dependent endophenotype will benefit from classifiers that are trained to predict specific endophenotypes, rather than classifiers that are nonspecific or agnostic.

We have recently developed an endophenotype prediction algorithm that trains endophenotype-specific cSNV classifiers [39]. The classifiers are, in part, a multiple-sequence alignment (MSA), the gene composition of which is optimized by iteratively maximizing the coefficient of determination (R-squared of regressing continuous-valued variables) between an internal score function and the cSNV-specific endophenotypes from the training set [39,48]. The score function considers amino acid conservation and the conservation of amino acid biophysical/biochemical properties, derived from the MSA. The score function can optionally consider 3-D structural data, as well. We refer to a classifier whose gene composition is optimized to predict an endophenotype as an endoPhenotype-Optimized Sequence Ensemble (ePOSE), and hence we call the method the ePOSE algorithm.

**Fig 4:**
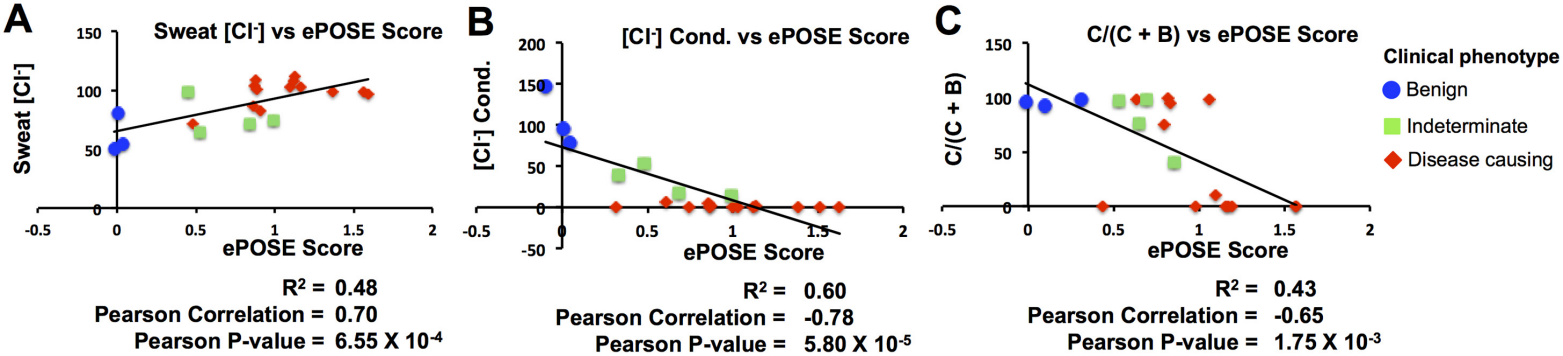
Correlation of ePOSE score with three individual endophenotypes. Measured endophenotype versus predicted impact (*ePOSE Score*) for 20 *CFTR* variants using classifiers trained with (A) sweat chloride, (B) chloride conductance, or (C) fraction of correctly processed CFTR protein. Each plot is the result of 20 leave-one-out cross-validation calculations (i.e., one data point for each of the 20 variants). Blue circles, green squares, and red diamonds denote benign, indeterminate, and disease-causing annotated phenotype, respectively, for each of the 20 variants. Note: increasing sweat chloride is associated with increasing disease severity, whereas for the two in vivo assays, decreasing values correspond to decreasing protein function or processing.

Fig 4 shows results from predicting three cystic fibrosis-related endophenotypes from 20 CFTR cSNVs (20 data points on each panel in Fig 4). For each of the three endophenotypes, individually, the ePOSE algorithm trained using 19 of 20 CFTR cSNVs, and prediction was made on the remaining cSNV; this process was repeated for each cSNV (leave-one-out cross-validation). Predictions were typically well correlated with the endophenotype being predicted, including reasonable separation of three clinically defined phenotypes associated with each cSNV (denoted by color and shape in Fig 4).

**Fig 5:**
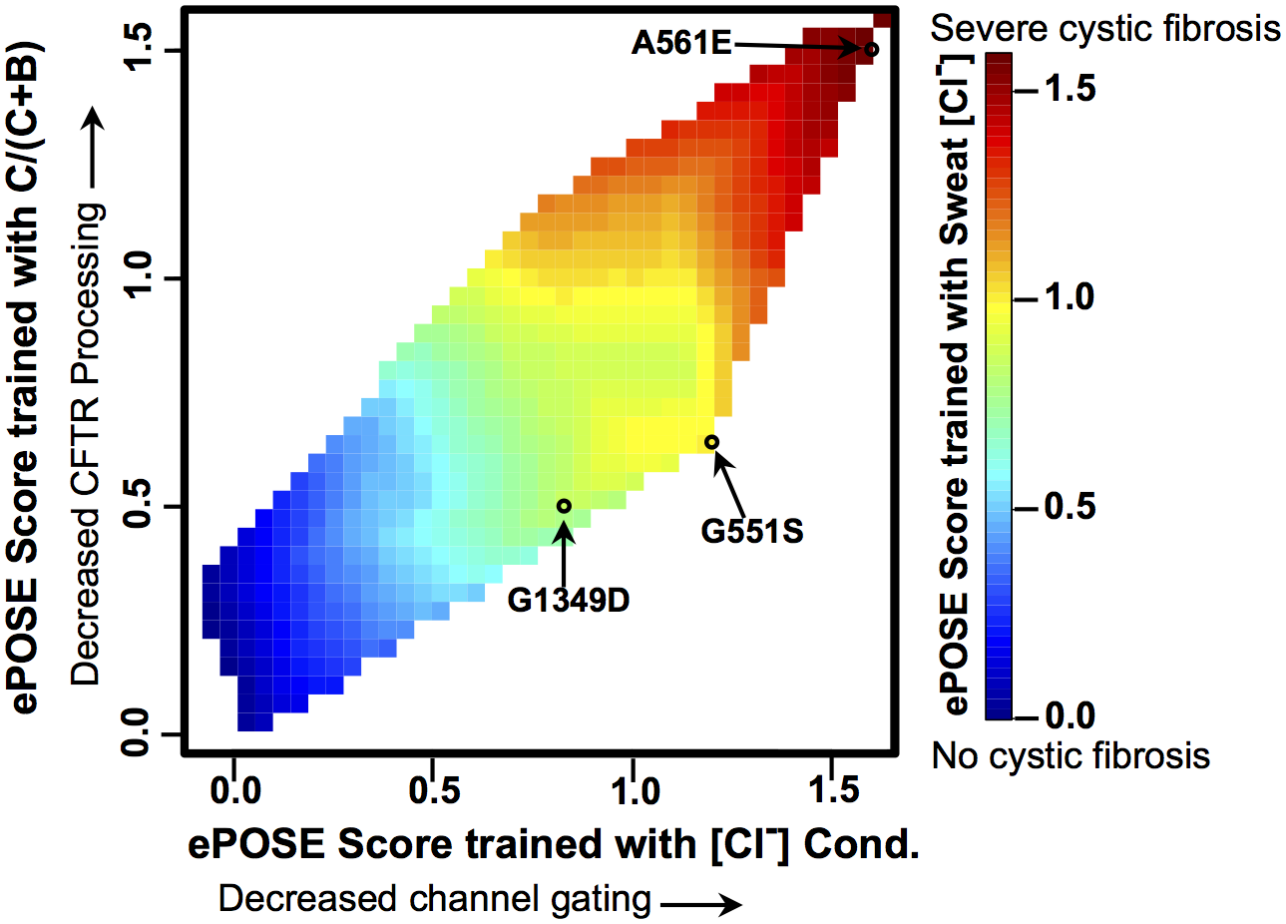
Interpolation plot of predicted endophenotypes resulting from the separate leave-one-out cross-validation calculations shown in Fig 4. ePOSE score for the 20 *CFTR* variants from Fig 4 plotted and interpolated (color shows ePOSE scores resulting from training with sweat chloride data). Using the resulting classifiers, each endophenotype was predicted for three additional variants (G551S, A561E, and G1349D) and subsequently validated. A561E was accurately predicted to affect disease via drastically reduced CFTR processing and channel gating. G551S was accurately predicted to affect cystic fibrosis primarily via channel gating.

In Fig 5, ePOSE predicts differential mechanisms associated with disease severity, including predictions for a validation set of three additional cSNVs, for which experimental and clinical data was collected prospectively. In contrast to Figs 3 and 5 is not hypothetical and shows actual ePOSE scores for each of three endophenotypes. The ePOSE algorithm accurately predicted that a significant fraction of the G551S cSNV would be processed and trafficked to the cell surface, but that chloride conductance would be significantly attenuated in cells expressing this cSNV. As described above, this same observation led to the development of Ivacaftor, a drug initially approved to target another G551 variant, G551D. Indeed, Ivacaftor has some efficacy for potentiating cells expressing the G551S cSNV as well [49].

Many of the existing in silico bioinformatic cSNV classifiers, originally designed to predict disease phenotypes, could be adapted to predict endophenotypes. Such efforts will require continued community-wide collection of data for algorithmic training and for independent benchmarking efforts. Locus-specific databases (LSDBs) already contain variant-specific endophenotypic information. Table 2 shows examples of training data currently available for endophenotypes associated with cystic fibrosis (CFTR), Li-Fraumeni and hereditary cancers (TP53) [50], phenylketonuria (PAH) [51], hypercholesterolemia and cardiovascular disease susceptibility (LDLR) [52], hereditary breast cancer (BRCA2) [53], and hyperhomocysteinemia (CBS) [54]. The CFTR2 database [40], which contains variants and endophenotypic data from ~40,000 cystic fibrosis patients, illustrates the potential of our suggested approach. For each patient, a reported genotype and up to six endophenotypes is provided. A mean value (and standard error) for an endophenotype of interest can be estimated, using reported values from patients with the same genotype (e.g., ~1,400 patients have one copy of the G551D allele and their mean sweat chloride is 104). Mean sweat chloride estimation is currently possible for ~250 unique variants in CFTR2, if a minimum of five patients with measured sweat chloride and sharing the identical allele is required [39].

**Table 2:** Six disease-associated genes with sources of variant-specific endophenotypic data.

Protein or gene (number of amino acids)	Endophenotype (number of unique mutations)	Disease phenotype(s)	References and database URLs
**CFTR (1,480)**	Sweat [Cl-] (>250)	Cystic fibrosis	[40]; cftr2.org
	FEV%1 (>250)		
	P.a. Infection (>250)		
	Pancreas function (>250)		
	C/ (C + B) (59)		
	[Cl-] Conductance (59)		
**TP53 (393)**	*WAF1* transactivation (2,314)	Li-Fraumeni and nonhereditary cancers	[50]; P53.iarc.fr
**PAH (452)**	Catalytic activity % wild type (80)	Phenylketonuria	[51]; www.pahdb.mcgill.ca
	PAH protein % wild type (80)		
	*PAH* mRNA % wild type (80)		
	Serum Phe and BH_4_ response (>35)		
**LDLR (860)**	Cellular LDL uptake (79)	Cardiovascular disease	[27,52]; umd.be/LDLR/
	Expression (79)	Hypercholesterolemia	ucl.ac.uk/ldlr
	Plasma LDL-c (~79)		
**BRCA2 (3,418)**	Homologous recombination (140)	Hereditary breast cancer	[53]
**CBS (551)**	Cell growth (204)	Hyperhomocysteinemia	[54]

Both locus-specific databases (LSDBs) and published manuscripts contain data to enable development of new in silico bioinformatics methods to predict variant impact on endophenotypes. For each gene, available endophenotypes, number of unique mutations with endophenotypic values, disease phenotype(s) and links/references to data sources is provided.

## Conclusion

As next-generation sequencing is integrated into routine patient care, in silico bioinformatic missense cSNV prediction tools have the potential to contribute to clinical practice. As of this writing, independent assessments of these tools indicate that they do not perform consistently, and there is considerable skepticism about their clinical utility. We reason that many of the apparent limitations are the result of a weakly defined paradigm. The tools are tasked with classifying cSNVs as disease causing, but most cSNVs by themselves do not have a large effect on whether an individual develops disease. The tools are also expected to assign cSNVs to phenotypic classes, although there is disagreement about how many of these classes should be considered and even which cSNVs belong in each class.

There are several potentially important, additional considerations regarding the performance of phenotypic prediction and what might be reasonably expected from endophenotypic prediction. First, many methods presented in Table 1 advertise the ability to predict variant impact on protein native state. While a connection between variant impact on protein native state and disease is often drawn, it is also acknowledged that these variables are not synonymous. Given that classifiers are often tasked with predicting impact on health or benchmarked using databases of putatively disease-causing variants, rather than assessing protein *damage*, methods are developed and challenged using disparate criteria. The Protein Mutant Database (PMD) employed in the Olatubosun et al. study [6] (Table 1) does record the impact of mutation on protein *activity*, potentially circumventing some of the above-described limitations. However, the PMD reduces continuous-valued activities (percent of wild type) to six discrete categories, and Olatubosun et al. further reduced categorization to either “functional” or “nonfunctional”; this clearly results in information loss, similar to that encountered when dichotomizing variants into discrete pathogenic and benign categories. It could be informative to compare the continuous-valued output from classifiers with the actual, non-stratified continuous-valued protein activities. This approach would be similar to that pursued in the PAH-PKU example from Wettstein et al. (above) [37]; this endophenotypic approach avoids the potentially dubious dichotomization of both algorithmic output and the experimental protein activities.

Endophenotype prediction presents new technical challenges, both in data acquisition and methods development. Although the large-scale development of gene-endophenotype databases will require community-wide effort, we see this as a tractable problem. Given that diseases are defined and diagnosed using quantitative endophenotypic risk factors, screens of genetic risk factors and association studies could, when possible, catalogue the continuous-valued endophenotypes used to partition the cases and controls. Some examples of this type of database curation are included in Table 2.

Wettstein et al. showed that some existing methods could potentially be repurposed for endophenotype prediction [37]. Even though most cSNV classifiers return dichotomous or categorical predictions, the underlying score functions calculate continuous-valued scores that could be correlated with measured endophenotypes. Although these existing classifiers suffer the limitation that they are not endophenotype specific, assessing the correlation of different endophenotypes and the continuous-valued output of existing methods could be informative. Also, the individual components of some methods’ score functions might be useful to help infer mechanism. For instance, SNPeffect combines four scores that each estimate distinct protein phenomena (amyloid formation, aggregation, stability, and chaperone binding) [55]. Classifiers that are gene and endophenotype specific—such as those produced by the ePOSE algorithm—will benefit from learning which variants (or genes) contribute to specific components of disease: as an example, CFTR variants that effect processing versus chloride conductance, or complex heart disease phenotypes that can result from varying combinations of *LDLR*-specific cholesterol plaques [27] or *LPA*-specific calcium plaques [56]. Undoubtedly, successful endophenotype prediction will benefit from diverse approaches.

Endophenotype predictors could be a useful complement for predicting complex diseases. A hallmark of complex disease is the presentation of varied combinations of traits associated with that disease, in which different traits can be influenced by different genetic risk factors. The quantitative traits are themselves endophenotypes, and predicting these objective traits, rather than a clinically defined abstraction of traits (i.e., phenotypes), could provide unique opportunities. For instance, predicting these quantitative traits facilitates the decomposition of complex disease into simpler, individual risk factors. For endophenotype predictors that are gene specific, this benefit will largely depend on a priori knowledge regarding causal genes.

In contrast to disease phenotype classes, endophenotypes are quantitative measurements having shared genetic underpinnings with disease phenotypes of interest. We suggest that in silico tools can be developed to predict the impact of cSNVs on endophenotypes, yielding improved accuracy and added value into the study of the mechanism and severity of cSNV impact on disease. The ePOSE algorithm provides a proof of concept and yields promising results in predicting three endophenotypes for a small set of cystic fibrosis cSNVs from the CFTR2 database [39]. The feasibility of such an approach will require community-wide efforts to augment the information currently available in LSDBs and other mutation databases.
